# Right versus Middle Hepatic Vein access and One-Year TIPS Outcomes

**DOI:** 10.1186/s42155-025-00570-x

**Published:** 2025-06-18

**Authors:** Vikrant Khare, Travis Merritt, Natalia Zbib, Linnea Swanson, Maria Masotti, Robert J. Fontana, Baljendra Kapoor, Hassan Anbari

**Affiliations:** 1 Vascular and Interventional Radiology, University of Michigan, 1500 E Medical Center Dr, Ann Arbor, MI 48109 USA; 2 Internal Medicine, University of Michigan, 1500 E Medical Center Dr, Ann Arbor, MI 48109 USA; 3 Department of Biostatistics in the School of Public Health, 1415 Washington Heights, Ann Arbor, MI 48109 USA

**Keywords:** Portal hypertension, Middle hepatic vein TIPS, Right hepatic vein TIPS, Hepatic encephalopathy, Portal hypertension related gastrointestinal hemorrhage

## Abstract

**Background:**

This study evaluates one-year clinical outcomes of transjugular intrahepatic portosystemic shunt (TIPS) placement using a middle hepatic vein (MHV) versus right hepatic vein (RHV) access. Primary end points were shunt patency and one-year survival. Secondary outcomes included incidence of de novo hepatic encephalopathy (HE) and recurrence of portal hypertension related complications such as ascites, hepatic hydrothorax, and gastrointestinal bleeding. While prior studies have examined portal vein target selection, the clinical relevance of hepatic vein choice remains understudied.

**Methods:**

A retrospective chart review of adult patients who underwent TIPS using a Viatorr stent graft between January 2014 and December 2022 was conducted. Patients were included if the procedure used either RHV or the MHV. Intracardiac echocardiography (ICE) was employed to select a direct path from hepatic to portal vein. Shunts were dilated to 8 or 10 mm to achieve a post-procedural portosystemic gradient (PSG) ≤ 12 mmHg or a 50% reduction from baseline. Clinical and imaging data was analyzed to assess outcomes, stratified by hepatic vein of access.

**Results:**

One-year survival (84% MHV vs 75% RHV, p = 0.2) and overall one-year patency rates (96% MHV vs 87% RHV, p = 0.5) were similar between the groups. However, MHV access significantly reduced de novo hepatic encephalopathy (30% MHV vs 62% RHV, p = 0.008) and moderate to severe cases (16% MHV vs 42% RHV, p = 0.017). Despite more frequent use of smaller diameter shunts (8 mm: 72% MHV vs 47% of RHV, p < 0.001), MHV access achieved similar post-TIPS portosystemic gradient reductions (Average Pre-TIPS gradient: 17 mmHg MHV & 17 mmHg RHV, p = 0.8; Average Post-TIPS gradient: 8 mmHg MHV & 7.5 mmHg RHV, p = 0.12). Hepatic vein choice did not affect outcomes for ascites, hydrothorax, or gastrointestinal bleeding.

**Conclusion:**

MHV and RHV access routes provided similar patency, survival, and TIPS indication outcomes, but MHV access had decreased incidence of hepatic encephalopathy and achieved similar portosystemic gradient reduction while using a smaller diameter shunt. MHV may be a preferred option for patients at higher risk of developing hepatic encephalopathy.

## Introduction

Transjugular intrahepatic portosystemic shunt (TIPS) reduces portal hypertension by creating a low resistance conduit within the liver between the portal and systemic circulation. It is used to treat portal hypertension complications such as variceal bleeding, refractory ascites, hepatic hydrothorax, Budd-Chiari syndrome, and portal vein thrombosis [1–5]. The volume of TIPS procedures has been stable but changes in aspects such as technique (e.g., use of ICE, transition from non-covered to covered stent grafts), patient selection (risk stratification of liver disease complications), and anatomic considerations (e.g., landing closer to the hepatocaval confluence) has led to improvement in the overall clinical parameters and long-term outcomes [1–15]. Historically, this has been performed by connecting the right hepatic vein (RHV) to the right portal vein (RPV). However, middle hepatic vein (MHV) and left hepatic vein (LHV) accesses, targeting various portions of the portal veins, are alternative approaches. Previous studies have demonstrated reduced overall incidence of post-TIPS hepatic encephalopathy (HE) when targeting the left portal vein (LPV) compared to the RPV [16–18]. The studies were conflicting with respect to differences in survival, rebleeding, recurrence or TIPS dysfunction – the largest of these studies showed no difference in these parameters [18]. To our knowledge, there’s no published study evaluating the hepatic vein choice of access and its impact on TIPS patency and clinical outcomes.

## Methods

### Clinical setting and study design

Following institutional review board approval and in accordance with HIPAA guidelines, a retrospective chart review study was conducted. The study reviewed medical records and imaging data from adult patients who had a Viatorr stentgraft (Viatorr; Gore, Flagstaff, Az, USA) placed between January 2014 to December 2022 from either the right or middle hepatic vein. Intracardiac echocardiography (ICE) (AccuNav; Accuson, Mountain View, CA) was used for selection of the hepatic and portal vein, accurate anatomic delineation, and used to guide the needle during the procedure to reduce procedure time and extracapsular puncture [13–15]. The Viatorr shunt was dilated to 8- or 10-mm diameter with a goal to achieve a post-procedural portosystemic gradient ≤ 12 mmHg or reduction of 50%. The hepatic and portal veins were deemed suitable if they could be visualized in a single plane using the ICE probe without intervening structures such as biliary ducts or other vasculature, and the decision to access the middle hepatic vein was based on the operator's discretion, prioritizing the most direct route into the portal system as observed on ICE. Stent patency and confirmation of the veins accessed was done using post-TIPS doppler. Inclusion and exclusion criteria are provided in Table 1.

**Table 1:** Inclusion and Exclusion criteria

*Inclusion criteria*	*Exclusion criteria*
Cirrhotic Liver disease	Non-cirrhotic portal HTN
Complete medical and imaging data available through 1 year of follow up	Age (< 18 years old)
Single VIATORR TIPS stent placed between JAN 2014 to DEC 2022	Liver transplant prior to TIPS placement
Right or middle hepatic vein access	Non-Viatorr stent graft or multi-stent TIPS
	Lack of follow up imaging after initial placement
	TIPS placed for prophylactic indications prior to surgery without overt decompensation

### Clinical outcomes

The primary outcome was shunt patency and one-year survival. Secondary outcomes were revision-free survival, efficacy of controlling gastrointestinal bleeding & fluid overload, and rate of new onset post-procedural HE.

In our study, post-TIPS HE was assessed using a novel classification system that stratifies patients based on the degree of intervention required. This grading system is as follows: 'None' for patients exhibiting no symptoms of HE; 'Mild' for cases necessitating the initiation of new pharmacologic therapy such as lactulose, rifaximin, or zinc – choice of medication was not standardized and was administered based on clinical judgement of the treating hepatologist, but was started at the first sign of altered mental status; 'Moderate' for patients requiring hospitalization in a non-ICU setting due to HE symptoms; and 'Severe' for cases where ICU admission or shunt reversal was warranted.

This customized stratification was chosen over the conventional West Haven classification system to provide a more clinically actionable framework. While the West Haven system remains a validated tool for HE severity assessment, our approach directly correlates HE severity with clinical resource utilization [19]. By focusing on the therapeutic and hospitalization needs, this system aligns with the practical considerations encountered in managing post-TIPS complications.

Clinical indications for TIPS placement included refractory fluid overload from hydrothorax or ascites that was not responsive to sodium restriction or diuretics necessitating recurrent thoracentesis or paracentesis. In addition, patients with refractory gastrointestinal bleeding requiring blood transfusions or repeated endoscopy were eligible for TIPS placement. Efficacy post-TIPS was categorized based on their management requirements after TIPS placement: absent, manageable without interventions (no thoracentesis, paracentesis, or transfusions required), manageable with sparing treatments, or refractory to standard treatments.

### Statistical Analysis

Statistical significance was established with a threshold of *p* < 0.05. The chi-square test was used for categorical variables, while continuous variables were analyzed using Wilcoxon rank sum test. For the estimation of revision-free survival, we treated death or revision as the endpoint and censored patients at transplant. Patients were followed from the procedure date until transplant, death, revision, or until loss to follow-up. Patients still being followed at 1-year post procedure were censored for analysis. Rates of revision free survival are compared via the log-rank test. Patency rates at one year post procedure are compared via Fisher’s exact test who were still being followed at one year. Cause-specific one-year survival for revision was estimated via cumulative incidence and compared via Gray’s test. Rates of clinical efficacy in the year post procedure were estimated via cumulative incidence and compared via Gray’s test. Rates of post-procedural HE in the first year were estimated via cumulative incidence and compared via Gray’s test.

## Results

### Patient Demographics

The study included 295 adult patients who underwent TIPS placement and compared those accessed via MHV to a RHV approach. The demographics and baseline clinical characteristics were essentially similar between the two groups as noted in Table 2.

**Table 2:** Baseline characteristics of the MHV and RHV groups, TIPS diameters, and portal vein accessed. Data presented as median (range) or number (%)

*Characteristics*	*MHV, N* = *57*	*RHV, N* = *238*	*P-Value*
*Age*	58 (50, 65)	59 (52, 66)	0.3
*Male (%)*	37 (65%)	141 (59%)	0.2
*Indication*			
*Ascites*	30 (52%)	145 (60%)	
*Hydrothorax*	3 (5%)	15 (6%)	
*Varices (esophageal and gastric)*	24 (42%)	80 (33%)	
*Pre-TIPS parameters* *MELD*	13.0 (10.0, 17.0)	12.0 (10.0, 17.0)	0.7
*Child-Turcotte-Pugh Score*	8.00 (7.00, 9.25)	8.00 (8.00, 9.00)	0.7
*CTP Class*	A: 6 (11%)	A: 14 (6.0%)	0.1
	B: 35 (63%)	B: 177 (76%)	
	C: 15 (27%)	C: 42 (18%)	
*Charlson Comorbidity Index*	5.00 (4.00, 6.00)	5.00 (4.00, 7.00)	0.2
*ALBI Score*	−1.76 (−2.22, −1.38)	−1.71 (−2.10, −1.29)	0.4
*Median Platelets (*× *10*^*3*^*/ml)*	87 (67, 152)	94 (64, 137)	0.7
*INR*	1.20 (1.10, 1.50)	1.20 (1.10, 1.40)	0.7
*Creatinine (mg/dl)*	0.90 (0.67, 1.40)	1.01 (0.78, 1.38)	0.2
*Albumin (g/l)*	3.20 (2.80, 3.70)	3.10 (2.70, 3.50)	0.2
*Total Bilirubin (mg/dl)*	1.50 (0.90, 2.80)	1.40 (0.90, 2.10)	0.4
*Sodium (meq/L)*	137.0 (133.0, 139.0)	136.0 (133.0, 139.0)	0.4
*Number of patients dilated to 8 or 10 mm TIPS Diameter (mm)*	8 mm: 41 (72%)	8 mm: 112 (47%)	** < 0.001**
	10 mm: 16 (28%)	10 mm: 126 (53%)	
*Average Pre-TIPS Gradient (mmHg)*	17 (14, 20)	17 (13, 20)	0.8
*Average Post-TIPS Gradient (mmHg)*	8 (6, 10)	7.5 (5, 10)	0.12
*Portal Vein Accessed*	Right: 29 (50%)	Right: 227 (95%)	
	Main: 17 (30%)	Main: 10 (4%)	
	Left: 11 (20%)	Left: 1 (1%)	

### One year patient Survival, Patency, and Clinical Outcomes

Clinical outcomes following TIPS placement in the two patient cohorts—MHV and RHV—were compared in this study (Table 3). One-year survival rates between MHV and RHV groups (84% vs 75%, *p* = 0.2) and revision rates (15% vs 17%, *p* = 0.7) were similar.

**Table 3:** Summary of clinical end points at one year. Data presented as median % (range)

*Outcome*		*MHV, N* = *57*	*RHV, N* = *238*	*p-value*
*Death*		16% (7.4%, 28%)	25% (20%, 32%)	0.2
*Revision*		15% (6.4%, 27%)	17% (12%, 22%)	0.7
*Revision Free Survival*	Varied^***1***^	69% (57%, 84%)	57% (51%, 65%)	0.16
*Rate of *de novo* hepatic encephalopathy*^***2***^	158 of 295	30% (12%, 49%)	62% (52%, 70%)	**0.008**
*Incidence of recurrence of hydrothorax*		8.5% (2.7%, 19%)	12% (7.5%, 17%)	0.6
*Incidence of recurrence of GI Bleeding*		2.2% (0.17%, 10%)	11% (6.3%, 16%)	0.12
*Incidence of recurrence of ascites*		30% (18%, 44%)	40% (33%, 48%)	0.3
*TIPS Patency*^***3***^		96%	87%	0.5

*(****1****) Under revision free survival, the total N is varied as this was a Kaplan–Meier curve (death and revision were failures, transplanted patients were censored). (****2****) Under rate of new hepatic encephalopathy, only patients with no episodes of previous hepatic encephalopathy were considered. (****3****) TIPS patency encompasses primary patency [no intervention], assisted primary patency [balloon angioplasty with or without stenting], and secondary patency [thrombectomy/thrombolysis with or without stenting] all at one year*

**Fig. 1:**
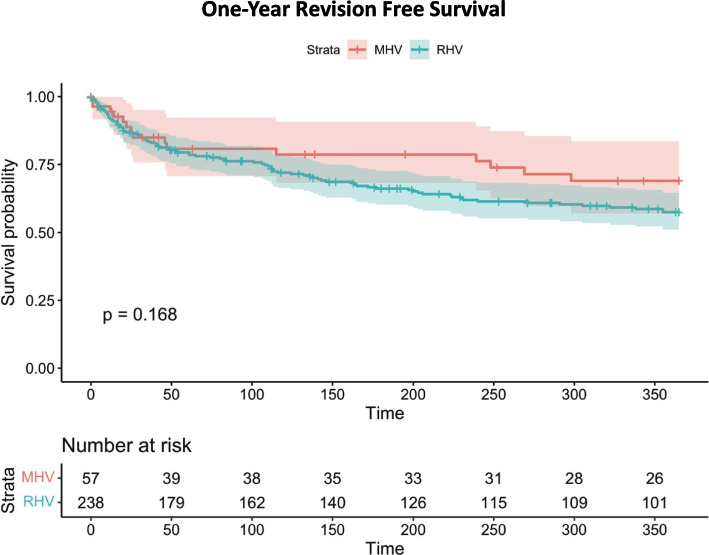
One year revision free survival. The x-axis shows time in days (over the period of one year) and the y-axis depicts the survival probability. Below the graph are the specific numbers in each group at intervals of 50 days

There was no statistically significant difference in the one-year revision-free survival rate between the MHV and RHV access (69% vs 57%, *p* = 0.16) (Fig. 1); this may be a trend that needs to be further studied but may not adequately powered for statistically significant analysis. TIPS patency and overall rate of revision were similar across both groups suggesting that either vein is an acceptable choice for access from a patency and rate of revision standpoint.

Clinical efficacy at one-year post-TIPS, defined as the degree of management required and/or improvement of hydrothorax, gastrointestinal bleeding, and ascites, showed no significant differences between the two groups, with *p*-values of 0.6, 0.3, and 0.12 respectively (Table 3).

**Fig. 2:**
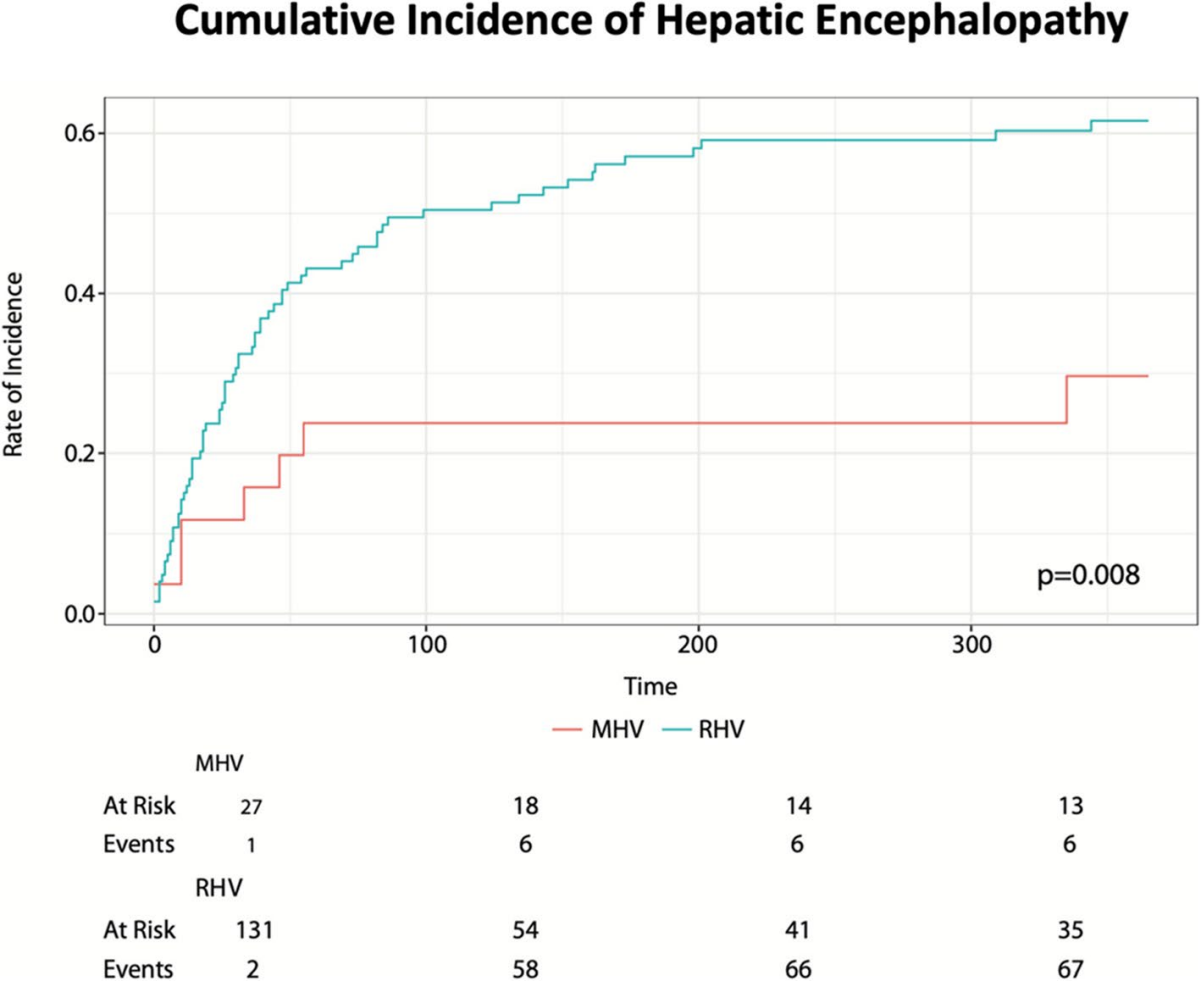
Cumulative incidence of hepatic encephalopathy at one year. The x-axis shows time in days (over the period of one year) and the y-axis shows the probability of hepatic encephalopathy within the population at risk at that given time; specific numbers are seen below the graph at 100 day intervals

**Fig. 3:**
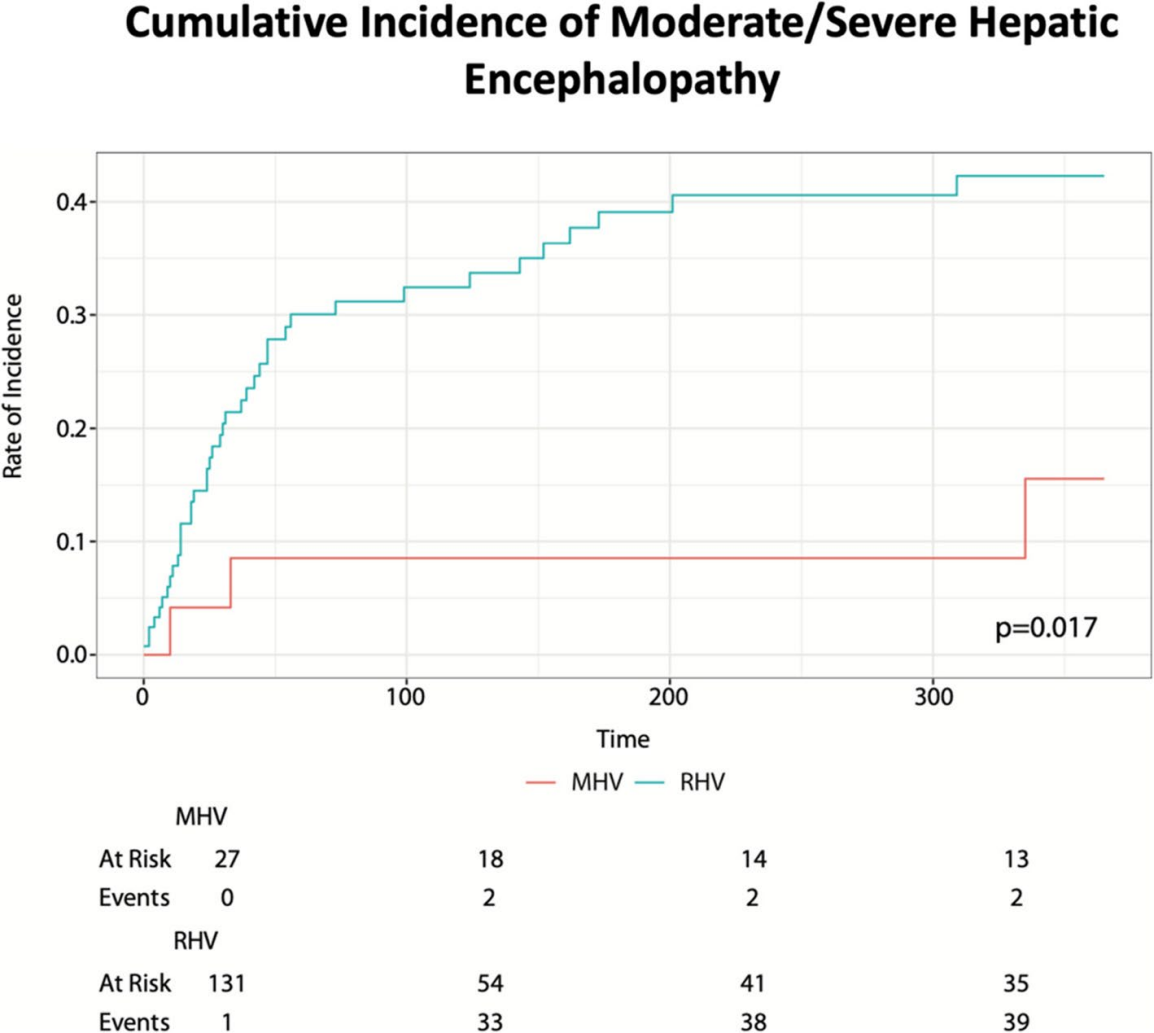
Cumulative incidence of moderate and severe hepatic encephalopathy. The x-axis shows time in days (over the period of one year) and the y-axis shows the probability of developing moderate-to-severe hepatic encephalopathy within the population at risk at that given time; specific numbers are seen below the graph at 100-day intervals

In a focused subgroup analysis of patients without pre-procedural HE, encompassing of 27 MHV and 131 RHV patients, post procedural HE was lower in the MHV group at 30% compared to 62% in the RHV group (***p* = 0.008**) as seen in Fig. 2. Furthermore, cumulative incidence of moderate and severe HE was lower in the MHV group at 16% compared to 42% in the RHV group (***p* = 0.017**) as seen in Fig. 3.

## Discussion

### Patency and survival

With respect to anatomic considerations of the procedure, TIPS terminating at the hepatocaval junction exhibit superior primary unassisted patency compared to those ending within the hepatic vein [12]. Building on this premise, this study explores whether the choice of hepatic vein—MHV versus RHV—affects shunt patency and clinical outcomes. Further investigation is warranted to determine whether initial vein selection can significantly impact patient outcomes, especially given that advances in patient selection, technique, and anatomic targeting have already been linked to improved clinical results [1–15].

Patency rates for TIPS over a 2 to 5-year follow-up period, including primary, primary-assisted, and secondary patency, inclusive of both covered and non-covered stents, have been reported between 72 to 93%, aligning with our study's results [1, 7–12]. In our cohort, TIPS primary and assisted patency was 96% for the MHV and 87% for the RHV, with revision rates of 15% and 17%, respectively.

We found no significant differences between MHV and RHV access in terms of overall patency rates—including primary, primary-assisted, and secondary patency—or revision-free survival. There were no observed differences in overall survival rates between the two access points. While our findings suggest that either access route is appropriate from a patency and survival standpoint, prospective randomized controlled trials are needed to confirm these results.

### Hepatic Encephalopathy, hydrothorax, ascites, and gastrointestinal hemorrhage

HE accounts for 25–45% of all major TIPS related complications [7, 8, 19]. Prior studies have highlighted a lower incidence of hepatic encephalopathy when the main portal vein was accessed, from either a right or middle hepatic access, compared to the right portal vein [16–18]. In the subgroup focused analysis, comprising of patients without pre-existing hepatic encephalopathy, we found a significantly lower post-procedural incidence of de novo HE in the MHV group (30% vs 62% for RHV, ***p* = 0.008**) and lower rates of moderate to severe HE (16% vs. 42% for RHV, ***p* = 0.017**). Our findings parallel studies studying left portal vein access, as in our population, the TIPS created from the middle hepatic vein were more often connected to the main or left portal vein (30% for MHV vs 4% for RHV) [16–18]. The difference in PV target may partially account for the observed reduction in HE incidence and is an important confounding factor; the observed benefits in the MHV group may reflect, at least in part, the PV that was chosen as the target.

The decrease in HE from a MHV approach is further confounded by the fact that 72% of the MHV TIPS were dilated to 8 mm compared to 47% in RHV group (***p* < 0.001**), as smaller TIPS are associated with lower rates of HE [20–22]. Despite this difference in shunt size, post-TIPS portosystemic gradients were similar between the two groups; these findings underscore the potential importance of initial vein selection, as the MHV group often required a smaller-diameter TIPS to achieve comparable outcomes. These findings are crucial, given the severe quality of life implications associated with HE and could guide pre-procedural planning and patient counseling [19–21, 23].

**Fig. 4:**
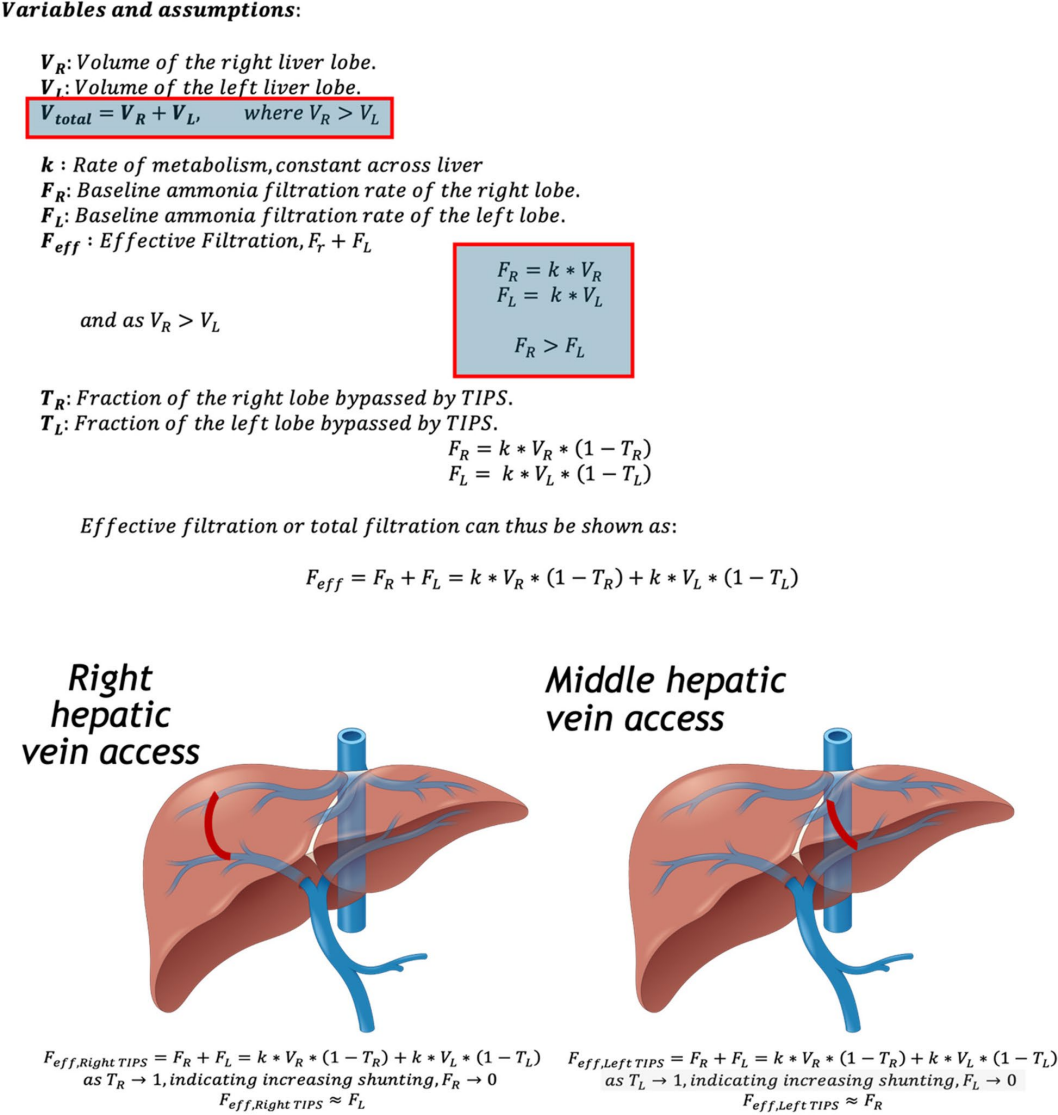
Effective Ammonia Filtration. If we assume complete bypass, a TIPS in each lobe, the respective lobe's contribution to ammonia filtration diminishes significantly. Since the right lobe is typically larger, its bypass has a more substantial impact on total ammonia filtration

Our study findings can be contextualized by a mathematical model focusing on ammonia filtration based on right versus left hepatic lobe volumes, seen in Fig. 4, and on blood-ammonia homeostasis within the portal vasculature [24, 25]. This model assumes that ammonia filtration is proportional to the volume of each lobe, and that TIPS would bypass that portion of the liver, thus reducing its effective filtration contribution. The MHV placement more frequently results in bypassing the left lobe and sparing the right lobe – which has a greater filtration capacity due to its larger volume – thus the overall reduction of the liver’s ammonia filtration capacity would be less [24, 25]. Computational filtration models exploring blood flow dynamics and its effect on ammonia filtration come to a similar conclusion [26].

There was no observed differences in the recurrence of the clinical indication for which TIPS was placed – namely gastrointestinal bleeding, ascites, or hydrothorax – when analyzed within the subgroup of patients who underwent TIPS for each respective indication (Table 3); this parallels previous studies where the accessed portal vein was the emphasis of the study [16–18]. Within these sub-groups, pre- and post- TIPS gradient changes were similar between the MHV and RHV groups. These findings suggest that ascites, hydrothorax, and gastrointestinal bleeding are reliant on overall changes in portosystemic gradient and are independent of shunt location. Thus, our findings indicate that similar clinical efficacy can be achieved for these indications with a smaller diameter shunt from a MHV approach.

### Study limitations

Despite the insights provided by this study, certain limitations should be acknowledged. The retrospective design introduces inherent biases and constrains generalizability of our study. A significant limitation was the unbalanced cohort sizes (MHV: 57, RHV: 238), which may have introduced skewed group-level variance and potentially masked small but clinically relevant differences. Selection bias likely influenced our results due to operator preference, given expected anatomic variability in hepatic vein sizes. Additionally, we could not account for all potential confounding variables affecting clinical outcomes, including operator variations in technique and post-procedural management protocols. These limitations highlight the need for prospective, controlled trials with larger, more balanced cohorts to validate our findings and establish definitive clinical recommendations regarding hepatic vein selection for TIPS procedure.

## Conclusion

Both middle hepatic and right hepatic vein TIPS access achieve high one-year patency rates and comparable survival outcomes. However, MHV access is associated with a significantly lower incidence of de novo hepatic encephalopathy (30% MHV vs 62% RHV, *p* = 0.008), particularly in moderate to severe cases (MHV 16% vs RHV 42%, *p* = 0.017). Importantly, in this subgroup, MHV access often required smaller-diameter shunts while achieving similar post-TIPS portosystemic gradient reductions.

Both MHV and RHV access routes demonstrated equivalent efficacy for managing ascites, hydrothorax, and gastrointestinal bleeding, achieving similar changes in PSG. Notably, MHV access accomplished these clinical outcomes using smaller diameter shunts (8 mm) while significantly reducing HE risk – without compromising TIPS effectiveness. This finding has important clinical implications, suggesting MHV may be the preferred approach for patients with higher hepatic encephalopathy risk. While our results showed comparable survival between access routes, previous research indicates that smaller TIPS diameters may confer survival benefits. The ability to achieve therapeutic goals with smaller goals represents a potential advantage of the MHV approach that warrants consideration in clinical practice [27].

## Data Availability

Data will be made on reasonable request. The datasets generated and/or analyzed during the current study are not publicly available due to **containing HIPAA-sensitive information** but are available from the corresponding author on reasonable request.

## Abbreviations

RHV: Right hepatic vein
MHV: Middle hepatic vein
LHV: Left hepatic vein
RPV: Right portal vein
MPV: Main portal vein
LPV: Left portal vein
PSG: Portosystemic gradient
HE: Hepatic encephalopathy
ICE: Intracardiac echocardiography

## Declarations

### SUBMISSION INFORMATION:

**Study Title**: A Review of the Safety and Efficacy of Transjugular Portosystemic Shunts in Patients with Chronic Liver Disease at the University of Michigan from 2005 to 2021.

### Full Study Title (if applicable):

**Study eResearch ID**: HUM00204528.

**Date of this Notification from IRB**: 5/11/2023.

**IRB Exemption Determination Date**: 5/11/2023.

**UM Federalwide Assurance**: FWA00004969 (For the current FWA expiration date, please visit the UM HRPP Webpage).

### EXEMPTION 4(iii) at 45 CFR 46.104(d):

**Secondary research for which consent is not required**: Secondary research uses of identifiable private information or identifiable biospecimens, if at least one of the following criteria is met:

**(iii)The research involves only** information collection and analysis involving the investigator's use of **identifiable health information when that use is regulated under** **45 CFR parts 160** **and 164, subparts A and E**, for the purposes of"health care operations"or"research"as those terms are defined at 45 CFR 164.501 or for"public health activities and purposes"as described under 45 CFR 164.512(b).

Note that the study is considered exempt as long as any changes to the use of human subjects (including their data) remain within the scope of the exemption category above. Any proposed changes that may exceed the scope of this category, or the approval conditions of any other non-IRB reviewing committees, must be submitted as another amendment through eResearch.

Although an exemption determination eliminates the need for ongoing IRB review and approval, you still have an obligation to understand and abide by generally accepted principles of responsible and ethical conduct of research. Examples of these principles can be found in the Belmont Report as well as in guidance from professional societies and scientific organizations.

### ACCESSING EXEMPT STUDIES IN eRESEARCH:

This study must now be accessed from the"Exempt and Not Regulated"tab of the eResearch Home Workspace. It no longer appears under the"Approved Studies"tab.

## References

[1] Suhocki P, Lungren M, Kapoor B, Kim C. Transjugular Intrahepatic Portosystemic Shunt Complications: Prevention and Management. Semin Interv Radiol. 2015May 28;32(02):123–32. 10.1055/s-0035-1549376 PMC4447874 26038620

[2] Mukund A, Aravind A, Jindal A, Tevethia HV, Patidar Y, Sarin SK. Predictors and Outcomes of Post-transjugular Intrahepatic Portosystemic Shunt Liver Failure in Patients with Cirrhosis. Dig Dis Sci. 2024Feb 10;69(3):1025–34. 38341393 10.1007/s10620-023-08256-x

[3] Vizzutti F, Schepis F, Arena U, Fanelli F, Gitto S, Aspite S, et al. Transjugular intrahepatic portosystemic shunt (TIPS): current indications and strategies to improve the outcomes. Intern Emerg Med. 2020Jan;15(1):37–48. 31919780 10.1007/s11739-019-02252-8

[4] Trivedi PS, Rochon PJ, Durham JD, Ryu RK. National Trends and Outcomes of Transjugular Intrahepatic Portosystemic Shunt Creation Using the Nationwide Inpatient Sample. J Vasc Interv Radiol. 2016Jun;27(6):838–45. 26965361 10.1016/j.jvir.2015.12.013

[5] Horhat A, Bureau C, Thabut D, Rudler M. Transjugular intrahepatic portosystemic shunt in patients with cirrhosis: Indications and posttransjugular intrahepatic portosystemic shunt complications in 2020. United European Gastroenterology Journal. 2021Feb 23;9(2):203–8. 32819214 10.1177/2050640620952637 PMC8259430

[6] T itton CM, Torikachvili M, Rêgo HMC, Medronha EF, Ziemiecki Junior E, Ribas C, et al. Transjugular intrahepatic portosystemic shunt in decompensated cirrhotic patients in a tertiary hospital in southern Brazil. Revista da Associação Médica Brasileira. 2023;69(4). 10.1590/1806-9282.20220944 PMC10176653 37075438

[7] Kraglund F, Jepsen P, Amanavicius N, Aagaard NK. Long-term effects and complications of the transjugular intrahepatic portosystemic shunt: a single-centre experience. Scand J Gastroenterol. 2019Jun 16;54(7):899–904. 31203699 10.1080/00365521.2019.1630675

[8] Ranjan Kumar Patel, Karamvir Chandel, Tara Prasad Tripathy, Amar Mukund. Complications of transjugular intrahepatic portosystemic shunt (TIPS) in the era of the stent graft – What the interventionists need to know? European journal of radiology. 2021 Nov 1;144:109986–6. 10.1016/j.ejrad.2021.109986 34619618

[9] Commins N, Subhaharan D, Kurup R, Wickremeratne T, Mitchell J, Elmes J, et al. Indications and outcomes of transjugular intrahepatic portosystemic shunt insertion in two regional Australian hepatology centres. Intern Med J. 2024Apr 23;54(8):1302–9. 38654627 10.1111/imj.16384

[10] Gaba RC, Omene BO, Podczerwinski ES, Knuttinen MG, Cotler SJ, Kallwitz ER, et al. TIPS for Treatment of Variceal Hemorrhage: Clinical Outcomes in 128 Patients at a Single Institution over a 12-Year Period. J Vasc Interv Radiol. 2012Feb;23(2):227–35. 22178037 10.1016/j.jvir.2011.10.015

[11] Weber CN, Nadolski GJ, White SB, Clark TWI, Mondschein JI, Stavropoulos SW, et al. Long-Term Patency and Clinical Analysis of Expanded Polytetrafluoroethylene-Covered Transjugular Intrahepatic Portosystemic Shunt Stent Grafts. Journal of vascular and interventional radiology : JVIR [Internet]. 2015 Sep;26(9):1257–65; quiz 1265. Available from: https://pubmed.ncbi.nlm.nih.gov/25990133/10.1016/j.jvir.2015.04.005 25990133

[12] Clark TWI, Agarwal R, Haskal ZJ, Stavropoulos SW. The Effect of Initial Shunt Outflow Position on Patency of Transjugular Intrahepatic Portosystemic Shunts. J Vasc Interv Radiol. 2004Feb;15(2):147–52. 14963180 10.1097/01.rvi.0000109401.52762.56

[13] Gipson MG, Smith MT, Durham JD, Brown A, Johnson T, Ray CE, et al. Intravascular US–Guided Portal Vein Access: Improved Procedural Metrics during TIPS Creation. J Vasc Interv Radiol. 2016Aug;27(8):1140–7. 26852944 10.1016/j.jvir.2015.12.002

[14] Dastmalchian S, Aryafar H, Tavri S. Intravascular Ultrasound Guidance for TIPS Procedures: A Review. Am J Roentgenol. 2022Oct;219(4):634–46. 35583424 10.2214/AJR.22.27626

[15] Ramaswamy RS, Charalel R, Guevara CJ, Tiwari T, Akinwande O, Kim SK, et al. Propensity-matched comparison of transjugular intrahepatic portosystemic shunt placement techniques: Intracardiac echocardiography (ICE) versus fluoroscopic guidance. Clin Imaging. 2019Sep;57:40–4. 31103908 10.1016/j.clinimag.2019.04.015

[16] Bai M. Shunting branch of portal vein and stent position predict survival after transjugular intrahepatic portosystemic shunt. World J Gastroenterol. 2014;20(3):774. 24574750 10.3748/wjg.v20.i3.774 PMC3921486

[17] Chen L, Xiao T, Chen W, Long Q, Li R, Fang D, et al. Outcomes of transjugular intrahepatic portosystemic shunt through the left branch vs. the right branch of the portal vein in advanced cirrhosis: a randomized trial. Liver International. 2009 Jul 2;29(7):1101–9. 10.1111/j.1478-3231.2009.02016.x 19386025

[18] Luo SH, Chu JG, Huang H, Zhao GR, Yao KC. Targeted puncture of left branch of intrahepatic portal vein in transjugular intrahepatic portosystemic shunt to reduce hepatic encephalopathy. World J Gastroenterol. 2019Mar 7;25(9):1088–99. 30862997 10.3748/wjg.v25.i9.1088 PMC6406189

[19] Weissenborn K. Hepatic Encephalopathy: Definition, Clinical Grading and Diagnostic Principles. Drugs. 2019Jan 31;79(S1):5–9. 30706420 10.1007/s40265-018-1018-z PMC6416238

[20] Liu J, Eric Paul Wehrenberg-Klee, Bethea ED, Uppot RN, Yamada K, Ganguli S. Transjugular Intrahepatic Portosystemic Shunt Placement for Portal Hypertension: Meta-Analysis of Safety and Efficacy of 8 mm vs. 10 mm Stents. Gastroenterology research and practice. 2020 Oct 17;2020:1–10. 10.1155/2020/9149065 PMC7586157 33123192

[21] Liu J, Ma J, Zhou C, Yang C, Huang S, Shi Q, et al. Potential Benefits of Underdilation of 8-mm Covered Stent in Transjugular Intrahepatic Portosystemic Shunt Creation. Clin Transl Gastroenterol. 2021Jun;12(6): e00376. 34140457 10.14309/ctg.0000000000000376 PMC8216680

[22] Cui J, Smolinski SE, Liu F, Xu D, Dulaimy K, Irani Z. Incrementally Expandable Transjugular Intrahepatic Portosystemic Shunts: Single-Center Experience. Am J Roentgenol. 2018Feb;210(2):438–46. 29261352 10.2214/AJR.17.18222

[23] Schindler P, Heinzow H, Trebicka J, Wildgruber M. Shunt-Induced Hepatic Encephalopathy in TIPS: Current Approaches and Clinical Challenges. Journal of Clinical Medicine [Internet]. 2020 Nov 23;9(11):3784. Available from: https://www.ncbi.nlm.nih.gov/pmc/articles/PMC7700586/10.3390/jcm9113784 PMC7700586 33238576

[24] Levitt D, Levitt M. A model of blood-ammonia homeostasis based on a quantitative analysis of nitrogen metabolism in the multiple organs involved in the production, catabolism, and excretion of ammonia in humans. Clin Exp Gastroenterol. 2018May;11:193–215. 29872332 10.2147/CEG.S160921 PMC5973424

[25] Damink SWMO, Jalan R, Redhead DN, Hayes PC, Deutz NEP, Soeters PB. Interorgan ammonia and amino acid metabolism in metabolically stable patients with cirrhosis and a TIPSS. Hepatology. 2002Nov;36(5):1163–71. 12395326 10.1053/jhep.2002.36497

[26] Yin K, Wang X, Zheng T. Computational hemodynamic analysis for optimal stent position in the transjugular intrahepatic portosystemic shunt procedure. J Biomech. 2022Oct;143: 111303. 36126502 10.1016/j.jbiomech.2022.111303

[27] Trebicka J, Bastgen D, Byrtus J, Praktiknjo M, Terstiegen S, Meyer C, et al. Smaller-Diameter Covered Transjugular Intrahepatic Portosystemic Shunt Stents Are Associated With Increased Survival. Clin Gastroenterol Hepatol. 2019Dec;17(13):2793-2799.e1. 30940552 10.1016/j.cgh.2019.03.042

